# Socioeconomic position and overweight among adolescents: data from birth cohort studies in Brazil and the UK

**DOI:** 10.1186/1471-2458-9-105

**Published:** 2009-04-15

**Authors:** Alicia Matijasevich, Cesar G Victora, Jean Golding, Fernando C Barros, Ana Maria Menezes, Cora L Araujo, George Davey Smith

**Affiliations:** 1 Post Graduate Programme in Epidemiology, Federal University of Pelotas, RS, Brazil; 2 Department of Community Based Medicine, University of Bristol, Bristol, England, UK; 3 Catholic University of Pelotas, RS, Brazil; 4 Department of Social Medicine, University of Bristol, Bristol, England, UK

## Abstract

**Background:**

Developed and developing countries are facing rapid increases in overweight and obesity among children and adolescents. The patterns of overweight/obesity differ by age, sex, rural or urban residence and socioeconomic position (SEP) and vary between and within countries.

**Methods:**

We investigated patterns of SEP – overweight status association among adolescents from the UK (ALSPAC) and Brazil (the 1982 and 1993 Pelotas birth cohort studies).

All analyses were performed separately for males and females. Logistic regression analysis was used to examine the relationships between overweight status and two SEP indicators – family income and maternal education.

**Results:**

A strong positive association was observed in 11-year-old boys from the 1993 Pelotas cohort, with higher prevalence of overweight among the least poor and among those whose mothers had more years of schooling (*x*^2 ^for linear trend p < 0.001). In ALSPAC study higher prevalence of overweight was seen among boys whose mothers had lower educational achievement (*x*^2 ^for linear trend p = 0.006). Among 11 year-old girls from 1993 Pelotas cohort study there was a positive association (higher prevalence of overweight in the higher socioeconomic and educational strata, *x*^2 ^for linear trend p < 0.001 and p = 0.01, respectively) while an inverse association was found in the ALSPAC study (*x*^2 ^for linear trend p < 0.001). Among males from the 1982 cohort study, overweight at 18 years of age showed a positive association with both SEP indicators while among females, the reverse association was found.

**Conclusion:**

The results of this study demonstrate that the social patterning of overweight varies between and within populations over time. Specific approaches should be developed within populations in order to contain the obesity epidemic and reduce disparities.

## Background

The association between socioeconomic position (SEP) and health is well established. McLaren [1] analyzed the socioeconomic patterning of weight across societies in various stages of socioeconomic development. In general, for both men and women, an increasing proportion of positive associations (higher SEP associated with smaller body size) and a decreasing proportion of negative associations (lower SEP associated with larger body size) was seen as one moved from countries with high levels to medium and low levels of socioeconomic development. In the process of economic development, changes for women seemed to take place before those for men.

There is a complex relationship between obesity and SEP in adults, because the direction of the association – whether obesity influences SEP or vice-versa – cannot be ascertained in cross-sectional studies. [2] Among children and adolescents, who as a rule do not contribute to their family's SEP, reverse causality is unlikely. Childhood SEP seems to be a more important determinant of body mass index (BMI) in adult life than current income [3], with several studies from high-income countries reporting an inverse association. [4-6]

High and middle income countries are facing rapid increases in overweight and obesity among children and adolescents. In England, obesity in children and adolescents of all ethnicities has increased nearly two-fold over the past 10 years.[7] In Brazil, the prevalence of obesity in older children and adolescents (aged 6–18 y) almost tripled between 1975 and 1997.[8] One of the reasons for concern about this trend is that overweight children and adolescents are likely to become fat adults. [9-12]

In the county of Avon, UK in 1991 and in the city of Pelotas, Brazil, in 1982 and 1993, population-based cohort studies were carried out providing the opportunity to investigate patterns of SEP-overweight status association among adolescents. The objectives of the present study were to investigate the association between overweight status and two indicators of SEP – family income and maternal education – among adolescents from the UK and Brazil, as well as to compare results between these populations.

## Methods

### Avon Longitudinal Study of Parents and Children (ALSPAC)

ALSPAC started during pregnancy and aimed to enrol all women living in the three Bristol-based health districts of the county of Avon and who had an expected date of delivery between April 1, 1991 and December 31, 1992. The study enrolled 14,541 pregnant women. Approximately 85% of the eligible mothers in the study have taken part. A detailed description of the methodology of this study is given elsewhere.[13] Pregnancy and perinatal data was obtained both from self-completion and interviewer applied questionnaires at various time points during pregnancy, as well as from clinical records. After birth, detailed information on growth has been collected from questionnaires sent to mother and their partner at least twice a year. From age 7 onwards all cohort subjects were invited to near-annual health checks. Of the 7159 adolescents who attended the clinic at 11 years of age (mean age of 11.8 y), anthropometric measurements were available for 6751 individuals (3341 boys and 3410 girls).

### Pelotas Birth Cohort Studies

The city of Pelotas, in Southern Brazil, has a population of about 330,000 inhabitants and more than 99% of all deliveries take place in hospitals. During the years of 1982 and 1993, birth cohort studies representing all births to mothers residing in the urban area of the city of Pelotas were carried out entailing primary data collection and using the same methodology. [14,15] Births were assessed by daily visits to all maternity hospitals, totalling 5914 newborns in 1982 and 4231 in 1993. Mothers were interview soon after delivery with a structured questionnaire and newborns were weighted and measured. Non-response rates at recruitment were below 1% in both cohorts. The cohort members were followed up at several points in time.

#### 1982 Pelotas cohort study

In 2000 and 2001 two follow-ups were carried out in the 1982 Pelotas cohort study. In 2000, male cohort adolescents (mean age 18.2 yrs) were identified at the time of attending the compulsory Army recruitment examination. A total of 2250 were interviewed (follow-up rate of 79%) and height and weight measurements were obtained for 2228 subjects. In 2001, a systematic sample of 27% of female cohort adolescents were visited at home (mean age 18.9 yrs). Anthropometric measurements were carried out on 919 women. The follow-up rate was 69%. Further information about these follow-ups can be found in a previous publication.[15]

#### 1993 Pelotas cohort study

In 2004–5 a follow-up of all adolescents born in 1993 was conducted (mean age 11.3 yrs). Weight and height were measured in the households by trained interviewers on 2161 boys and 2280 girls with a follow-up rate of 87.5%. A detailed description of the methodology of this study is given elsewhere.[14]

### Definitions of overweight and obesity

Weight and height measurements were performed at each follow-up in each cohort study by trained interviewers with children or adolescents dressed in underwear and barefoot. Body mass index (BMI) was calculated as weight in kilograms divided by height in meters squared. According to the WHO BMI-for-age and sex reference charts [16] overweight and obesity were defined as BMI-for-age > + 1 SD and > + 2 SD respectively, according to age in months and sex.

### Other data

Gender was obtained from the perinatal data in both ALSPAC and Pelotas birth cohort studies. Subject's age was calculated from the date of birth and the date of attendance at the research clinic in ALSPAC study and the date of interview in the Pelotas cohort studies.

Two SEP indicators were used: family income and maternal education. In ALSPAC, family income per week was collected at 33 months after delivery. In the 1982 and 1993 Pelotas cohort studies family income of the month prior to the interview was collected in the perinatal interview. To allow comparisons between studies, quintiles of family income were calculated in each study. In the 1993 Pelotas cohort study, family income was collected as a continuous variable (in Reales) and quintiles were calculated directly. In ALSPAC and in the 1982 Pelotas cohort study, family income was collected as a grouped variable. A principal components analysis was carried out in ALSPAC and the 1982 Pelotas cohort studies and the first component was used to derive a score that was used to rank individuals within family income groups. Cut-off points were then found within each category so that five nearly equal sized groups were formed in each cohort. [17]

Maternal schooling at the time of delivery was categorised in 0 to 4, 5 to 8 and = 9 completed years of formal education in the Pelotas cohort studies. In ALSPAC study, maternal education was assessed at 32 weeks of gestation and categorised in three groups according to increasing levels of achievement: CSE/vocational, O-level and A-level/degree.

### Statistical analysis

The comparison between ALSPAC and 1993 Pelotas cohort study refers to the age of 11 years. To test whether the association of SEP indicators with overweight varied by sex, tests for statistical interaction were performed in each cohort study. There was a suggestion that interactions existed in each case (p value range 0.051 to 0.003) and therefore analyses were performed separately for males and females.

Logistic regression analysis was used to examine the relationship between overweight status and the two SEP indicators. In addition, to test the independent effects of maternal education and family income, each SEP was adjusted for the other indicator and for height in a separate analysis.

Statistical significance was defined as a two-tailed p-value below 0.05. All analyses were performed using STATA^® ^version 10.0 (StataCorp LP, College Station, Texas, USA).

### Details of Ethics Approval

Ethical approval for the study was obtained from the ALSPAC Law and Ethics Committee and the Local Research Ethics Committees. The study protocol of 1982 and 1993 Pelotas cohort studies was approved by the Medical Ethics Committee of the Federal University of Pelotas, affiliated with the Brazilian Federal Medical Council.

## Results

### Cohort characteristics

Baseline characteristics of the study participants by cohort study and sex are shown in Table 1 ("See additional file 1: Descriptive characteristics of ALSPAC and Pelotas 1982 and 1993 populations according to sex").

While no gender differences in maternal education were observed in ALSPAC and the 1993 Pelotas studies, females from the the 1982 Pelotas cohort were more likely to have mothers with fewer years of schooling. No gender differences in mean age at follow-up were seen in ALSPAC and the 1993 Pelotas cohort studies. In the 1982 Pelotas cohort, women were older than men because their follow-up was carried out one year after males' follow-up. Boys had higher prevalence of obesity and overweight than girls in ALSPAC and in the 1993 Pelotas cohort study. No gender differences were observed in the prevalence of overweight or obesity in the 1982 Pelotas cohort study.

Boys and girls from ALSPAC study were older than those from 1993 Pelotas cohort study (Students's *t*-test p < 0.001, for each gender). Girls from ALSPAC had higher mean BMI than those from the 1993 Pelotas cohort: 19.4 (SD 3.5) and 18.6 (SD 3.6) kg/m^2^, respectively (Students's *t*-test p < 0.001). A smaller difference was found between boys, who had mean BMIs of 18.8 (SD 3.3) and 18.6 (SD 3.5) kg/m^2 ^in ALSPAC and 1993 Pelotas cohort study, respectively (Students's *t*-test p = 0.06). Overweight prevalence rate (BMI-for-age >+ 1SD) was almost the same among boys from ALSPAC and the 1993 Pelotas study (31.5% and 30.8%, respectively, *x*^2 ^test p = 0.7). Girls from ALSPAC showed higher prevalence of overweight than their peers from the 1993 Pelotas cohort (29.9% and 26.5%, respectively, *x*^2 ^test p = 0.011). Prevalence of obesity (BMI-FOR-AGE>+ 2SD) was lower among boys from ALSPAC study than those from the 1993 Pelotas cohort (10.3% and 12.3%, respectively, *x*^2 ^test p = 0.01), but no difference was seen between girls (8.7% and 8.2%, respectively, *x*^2 ^test p = 0.8).

### Overweight and obesity rates according to SEP

**Figure 1:**
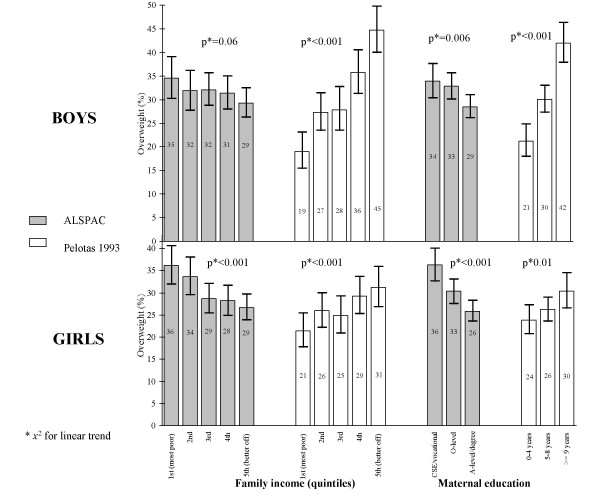
**Distribution of overweight prevalence rates by SEP among 11 year-olds, ALSPAC and 1993 Pelotas cohorts**. Overweight: Body mass index > 1 standard deviation according to WHO's growth charts of BMI-for-age and sex (obese children included); SEP: socioeconomic position indicator (family income and maternal education).

The distribution of overweight prevalence rates and 95% confidence intervals among 11 year-old adolescents by sex and SEP indicators adjusted for age is shown in Figure 1. A strong positive association was observed in 11-year-old boys from the 1993 Pelotas cohort study, with higher prevalence of overweight among the least poor and among those whose mothers had more years of schooling. In ALSPAC higher prevalence of overweight was seen among boys whose mothers had lower educational achievement but no robust association was found between overweight and family income. Among 11 year-old girls overweight was associated with both SEP indicators. However, while in the 1993 Pelotas cohort study there was a positive association (higher prevalence of overweight in the higher socioeconomic and educational strata), an inverse association (higher prevalence of overweight among the poorest and among daughters of women with the lowest educational achievement) was found in ALSPAC.

**Figure 2:**
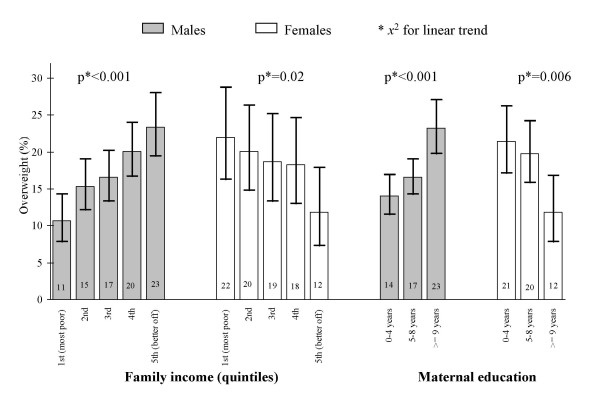
**Distribution of overweight prevalence rates by SEP among 18 year-olds, 1982 Pelotas cohort**. Overweight: Body mass index > 1 standard deviation according to WHO's growth charts of BMI-for-age and sex (obese adolescents included); SEP: socioeconomic position indicator (family income and maternal education).

In the 1982 cohort study, overweight at 18 years of age was associated with both SEP indicators, but different patterns of association were seen among males and females. Among males a positive association (higher prevalence of overweight in higher socioeconomic and educational strata) was seen while among females, the reverse association was found (Figure 2).

### Multivariate analyses

Among males from the ALSPAC study, the association between overweight and maternal education remained virtually unchanged after adjustment for family income. No association was found between overweight and family income, neither in the crude nor in the adjusted analysis (Table 2, "See additional file 2: Crude and adjusted association between SEP indicators and overweight in each cohort study"). Among girls, maternal education indicator showed independent effects on overweight in the adjusted analysis.

In the 1993 Pelotas cohort study, adolescents from higher socioeconomic and educational strata were more likely to be overweight. Among males, the association between overweight and both SEP indicators was maintained in the adjusted analysis, even though the magnitude of the ORs was reduced. Among girls, adjusting for family income removed the differentials in the association between overweight and maternal education. The association between overweight and family income was also removed after adjustment for maternal education.

In the 1982 Pelotas cohort study, 18 year-old men from higher socioeconomic strata and sons of women with higher educational achievement showed higher risk of overweight in the crude analysis. The association between overweight and maternal education disappeared in the adjusted analysis, while the association between overweight and family income remained. Among females, the crude analyses showed an association between overweight and maternal education, which was attenuated after adjustment.

## Discussion

Our analyses show that overweight is related to SEP indicators, but that relationship differs between populations and within populations over time. In this study, the association between overweight and SEP indicators should not be influenced by reverse causality, as can occur in adults,[18] because SEP was determined by parents' characteristics and information about SEP indicators was collected in early childhood or at birth.

Girls from ALSPAC had higher mean BMI and higher overweight rates than those from the 1993 Pelotas cohort study. Early sexual maturation is one factor consistently related to increased body weight in adolescents and young adults.[19-21] Median age at menarche in girls from the 1993 Pelotas cohort study was assessed at the age of 15 years (after the visit reported in this paper) and was slightly below the reported median age at menarche of British girls [22] (12.3 *vs *12.9 years for Pelotas and British teenagers, respectively). Unfortunately, pubertal state was not assessed in the Pelotas cohort studies preventing us to explain differences in mean BMI and overweight rates by differences in onset of puberty in ALSPAC and Pelotas populations.

In the ALSPAC study, female adolescents from high income and educational strata had a lower prevalence of overweight than their counterparts, a pattern that is consistent with findings from high-income countries.[23-25] In the Pelotas study, however, boys and girls at 11 years of age from high income and educational strata were at risk of having the highest prevalence of overweight, results that are in accordance with findings from low and middle-income country settings.[23,24]

In the Pelotas cohort by age 18 gender became an effect modifier in the association between overweight and both SEP indicators; there was a direct association in males and an inverse association in females. Wang [24] showed similar results with data from Russia and China.

The way in which paternal SEP could influence their adolescents' overweight status is complex.[2] Adolescent's home and school environments, as well as parenting styles have been associated with patterns of eating and physical activity.[26,27] Patterns of low energy expenditure among the richest and cultural values favouring a large body size, especially among children, could have contributed to the positive association between overweight and SEP indicators observed in the Pelotas cohort study at 11 years of age.

A different scenario for the relationship between overweight and SEP indicators emerged among adolescents at 18 years of age. In the Brazilian culture, society's pressure toward thinness, particularly among women and adolescent females is strong. [28] It is possible that better-off mothers or with more years of schooling would be concerned that their daughters would become overweight and have to face the stigmatization of obesity, leading to greater monitoring of girls' behaviours than boys'. Monteiro et al.[29] reported that the burden of adult obesity in the developing world tends to shift towards the groups with lower SEP as the level of economic development increases.

## Conclusion

The results of this study reinforce the contention that the social patterning of BMI varies between populations and within populations over time. Strategies for obesity prevention should not be restricted to particular SEP, gender or age groups, but tailored approaches should be developed within populations in order to contain the obesity epidemic and reduce disparities.

## Competing interests

The authors declare that they have no competing interests.

## Authors' contributions

AM and GS originated the research question. AM conducted the analyses and wrote the first draft of the article. GS, JG and CV supervised the analysis and interpretation of the findings, as well as the writing of the article. FB, AMM and CA contributed to the interpretation of the analyses and assisted with the editing of the article. All authors read and approved the final manuscript.

## Pre-publication history

The pre-publication history for this paper can be accessed here:



## Supplementary Material

Additional file 1 **Table 1: Descriptive characteristics of ALSPAC and Pelotas 1982 and 1993 populations according to sex.** Click here for file

Additional file 2 **Table 2: Crude and adjusted association between SEP indicators and overweight (BMI >1 SD for gender and age), ALSPAC and Pelotas 1982 and 1993 cohort studies.** Click here for file
