# Supplementary material for: Socioeconomic position and overweight among adolescents: data from birth cohort studies in Brazil and the UK
Source: BMC Public Health. 2009 Apr 15;9:105. doi: 10.1186/1471-2458-9-105 (PMC2673220; doi:10.1186/1471-2458-9-105)
Supplement: Additional file 1 — Table 1: Descriptive characteristics of ALSPAC and Pelotas 1982 and 1993 populations according to sex. [file 1471-2458-9-105-S1.doc]

Table 1. Descriptive characteristics of ALSPAC and Pelotas 1982 and 1993 populations according to sex.

| Variables | ALSPAC | | | | Pelotas 1993 cohort | | | | | Pelotas 1982 cohort | | | | |
| --- | --- | --- | --- | --- | --- | --- | --- | --- | --- | --- | --- | --- | --- | --- |
|  | Males | Females | | p | Males | | | Females | p | Males | | | Females | p |
| *Variables at birth* | | | | | | | | | | | | | | |
| Maternal education (years), n (%)  0-4  5-8  ≥ 9  Maternal education (achievement)^a^, n (%)  CSE/vocational  O-level  A-level/degree | 689 (21.9)  1117 (35.6)  1335 (42.5) | 691 (21.6)  1137 (35.5)  1371 (42.9) | | 0.938^d^ | 571 (26.5)  1046 (48.5)  540 (25.0)  -  -  - | | 658 (28.9)  1076 (47.3)  543 (23.9)  -  -  - | | 0.188 ^d^ | | 691 (31.1)  982 (44.2)  551 (24.8)  -  -  - | | 332 (36.2)  365 (39.8)  221 (24.0)  -  -  - | 0.016 ^d^ |
| Family income (quintiles) ^b^, n (%)  1^st^ (most poor)  2^nd^  3^rd^  4^th^  5^th^ (better off) | 463 (14.2)  474 (14.6)  744 (22.9)  702 (21.6)  868 (26.7) | 503 (15.1)  490 (14.7)  738 (22.2)  700 (21.1)  893 (26.9) | | 0.828 ^d^ | 430 (19.9)  511 (23.7)  383 (17.7)  429 (19.9)  408 (18.9) | | 453 (19.9)  500 (21.9)  422 (18.5)  481 (21.1)  424 (18.6) | | 0.609 ^d^ | | 374 (16.8)  457 (20.5)  477 (21.4)  487 (21.9)  433 (19.4) | | 186 (20.2)  204 (22.2)  182 (19.8)  186 (20.2)  161 (17.5) | 0.088 ^d^ |
| *Variables at the follow-up of interest* | | | | | | | | | | | | | | |
| Age at follow-up (years), mean (sd),  minimum and maximum value | 11.7 (0.2)  10.7-13.5 | 11.7 (0.2)  10.4-13.6 | 0.602^e^ | | 11.3 (0.3)  10.6-12.2 | 11.3 (0.3)  10.6-12.2 | | | 0.867 ^e^ | | | 18.2 (0.3)  17.6-19.1 | 18.9 (0.3)  18.1-19.8 | <0.001 ^e^ |
| BMI at follow-up (kg/m^2^), mean (sd) | 18.8 (3.3) | 19.4 (3.5) | <0.001^e^ | | 18.6 (3.5) | 18.6 (3.6) | | | 0.935 ^e^ | | | 22.3 (3.7) | 22.5 (4.4) | 0.237 ^e^ |
| Overweight ^c^ , n (%) | 1029 (31.5) | 1001 (29.9) | 0.163^d^ | | 666 (30.8) | 606 (26.5) | | | 0.002^d^ | | | 388 (17.4) | 169 (18.4) | 0.515 ^d^ |
| Obesity ^c^, n (%) | 337 (10.3) | 292 (8.7) | 0.027^d^ | | 267 (12.3) | 187 (8.2) | | | <0.001^d^ | | | 113 (5.1) | 50 (5.4) | 0.671^d^ |
| Number of individuals | 3341 | 3410 | - | | 2161 | 2280 | | | - | | | 2250 | 919 | - |

^a^ variable collected at 32 weeks of pregnancy in ALSPAC study

^b^ variable collected at 33 months after delivery in ALSPAC study

^c^ according to WHO’s growth charts of BMI-for-age and sex

^d^ *x*^2^ test

^e^ Students´s *t*-test
