# Supplementary material for: Socioeconomic position and overweight among adolescents: data from birth cohort studies in Brazil and the UK
Source: BMC Public Health. 2009 Apr 15;9:105. doi: 10.1186/1471-2458-9-105 (PMC2673220; doi:10.1186/1471-2458-9-105)
Supplement: Additional file 2 — Table 2: Crude and adjusted association between SEP indicators and overweight (BMI >1 SD for gender and age), ALSPAC and Pelotas 1982 and 1993 cohort studies. [file 1471-2458-9-105-S2.doc]

Table 2. Crude and adjusted association between SEP indicators and overweight (BMI >1 SD for gender and age)^a^ in each cohort study.

| SEP indicators | ALSPAC | | 1993 Pelotas cohort study | | 1982 Pelotas cohort study | | |
| --- | --- | --- | --- | --- | --- | --- | --- |
|  | Males  OR (IC 95%) | Females  OR (IC 95%) | Males  OR (IC 95%) | Females  OR (IC 95%) | Males  OR (IC 95%) | Females  OR (IC 95%) | |
| *Crude analyses* | | | | | | | |
| Maternal education (years)  0-4  5-8  ≥ 9  Maternal education (achievement)  CSE/vocational  O-level  A-level/degree | -  -  -  *p=0.016*  Reference  0.95 (0.78; 1.16)  0.78 (0.64; 0.95) | -  -  -  *p<0.001*  Reference  0.76 (0.63; 0.93)  0.61 (0.50; 0.74) | *p<0.001*  Reference  1.59 (1.25; 2.03)  2.69 (2.07; 3.50)  -  -  - | *p=0.036*  Reference  1.13 (0.90; 1.42)  1.40 (1.08; 1.80)  -  -  - | *p<0.001*  Reference  1.22 (0.93; 1.60)  1.85 (1.38; 2.48)  -  -  - | | *p=0.009*  Reference  0.90 (0.63; 1.30)  0.49 (0.30; 0.80)  -  -  - |
| Family income (quintiles)  1^st^ (poorest)  2^nd^  3^rd^  4^th^  5^th^ (better off) | *p=0.400*  Reference  0.89 (0.68; 1.17)  0.90 (0.70; 1.15)  0.87 (0.68; 1.12)  0.79 (0.62; 1.00) | *p<0.001*  Reference  0.89 (0.69; 1.16)  0.71 (0.56; 0.90)  0.69 (0.54; 0.88)  0.64 (0.51; 0;81) | *p<0.001*  Reference  1.60 (1.18; 2.18)  1.65 (1.19; 2.29)  2.37 (1.74; 3.24)  3.45 (2.53; 4.70) | *p=0.008*  Reference  1.29 (0.95; 1.74)  1.22 (0.89; 1.66)  1.53 (1.13; 2.06)  1.67 (1.23; 2.26) | *p<0.001*  Reference  1.51 (1.00; 2.29)  1.66 (1.10; 2.49)  2.10 (1.42; 3.12)  2.54 (1.71; 3.78) | | *p=0.127*  Reference  0.89 (0.55; 1.44)  0.81 (0.49; 1.35)  0.79 (0.48; 1.32)  0.47 (0.26; 0.85) |
| *Analyses adjusted for the other SEP indicator and height* | | | | | | | |
| Maternal education (years)  0-4  5-8  ≥ 9  Maternal education (achievement)  CSE/vocational  O-level  A-level/degree | *p=0.004*  Reference  0.95 (0.77; 1.18)  0.72 (0.58; 0.90) | *p<0.001*  Reference  0.78 (0.63; 0.95)  0.63 (0.50; 0.78) | *p=0.010*  Reference  1.32 (1.02; 1.70)  1.57 (1.16; 2.13)  -  -  - | *p=0.839*  Reference  0.94 (0.74; 1.20)  0.92 (0.68; 1.24)  -  -  - | *p=0.360*  Reference  1.06 (0.79; 1.42)  1.31 (0.89; 1.93) | | *p=0.150*  Reference  0.92 (0.61; 1.39)  0.54 (0.28; 1.04) |
| Family income (quintiles)  1^st^ (poorest)  2^nd^  3^rd^  4^th^  5^th^ (better off) | *p=0.385*  Reference  0.79 (0.59; 1.07)  0.91 (0.70; 1.19)  0.79 (0.60; 1.04)  0.82 (0.63; 1.08) | *p=0.095*  Reference  0.92 (0.70; 1.22)  0.77 (0.60; 0.99)  0.75 (0.57; 0.97)  0.73 (0.56; 0.95) | *p=0.002*  Reference  1.27 (0.91; 1.76)  1.17 (0.82; 1.66)  1.54 (1.09; 2.15)  1.94 (1.36; 2.77) | *p=0.607*  Reference  1.19 (0.87; 1.63)  1.09 (0.78; 1.53)  1.27 (0.92; 1.76)  1.24 (0.87; 1.76) | *p=0.025*  Reference  1.49 (0.98; 2.26)  1.61 (1.06; 2.46)  1.93 (1.26; 2.97)  2.12 (1.30; 3.46) | | *p=0.844*  Reference  0.90 (0.55; 1.46)  0.84 (0.48; 1.45)  0.92 (0.51; 1.63)  0.66 (0.31; 1.54) |

^a^ According to WHO’s growth charts of BMI-for-age and sex
